# ﻿A hotspot of lichen diversity and lichenological research in the Alps: the Paneveggio-Pale di San Martino Natural Park (Italy)

**DOI:** 10.3897/mycokeys.94.95858

**Published:** 2022-12-06

**Authors:** Juri Nascimbene, Gabriele Gheza, Peter O. Bilovitz, Luana Francesconi, Josef Hafellner, Helmut Mayrhofer, Maurizio Salvadori, Chiara Vallese, Pier Luigi Nimis

**Affiliations:** 1 BIOME Lab, Department of Biological, Geological and Environmental Sciences, Alma Mater Studiorum University of Bologna, Via Irnerio 42, 40126 Bologna, Italy Alma Mater Studiorum University of Bologna Bologna Italy; 2 Division of Plant Sciences, Institute of Biology, NAWI Graz, University of Graz, Holteigasse 6, 8010 Graz, Austria University of Graz Graz Austria; 3 Paneveggio-Pale di San Martino Natural Park, Villa Welsperg, località Castelpietra 2, 38054 Primiero San Martino di Castrozza (Trento), Italy Paneveggio-Pale di San Martino Natural Park Primiero San Martino di Castrozza Italy; 4 Department of Life Sciences, University of Trieste, Via L. Giorgieri 10, 34127 Trieste, Italy University of Trieste Trieste Italy

**Keywords:** Alps, biodiversity, checklists, conservation, herbarium studies, historical records, lichen inventories

## Abstract

A checklist of 916 lichenised taxa is reported from the Paneveggio-Pale di San Martino Natural Park and its surroundings (Trentino-Alto Adige, N Italy), based on 7351 records from: (a) 72 literature sources, (b) eight public and private herbaria and (c) field observations by some of the authors. The study area appears as a hotspot of lichen diversity, hosting 30.1% of the lichen biota of the Alps in a territory that has 0.064% of their total surface area. This is mainly due to its high climatical, geological and orographic heterogeneity, but also to the long history of lichenological exploration, that started in the 19^th^ century with Ferdinand Arnold and is still ongoing. The present work highlights the importance of detailed species inventories to support knowledge of biodiversity patterns, taxonomy and ecology and to properly address conservation issues. Fuscideamollisvar.caesioalbescens, *Hydropunctariascabra*, Protoparmeliabadiavar.cinereobadia and *Variosporapaulii* are new to Italy, 18 other taxa are new to Trentino-Alto Adige.

## ﻿Introduction

Basic information on the distribution, ecology and taxonomy of species is fundamental for revealing biodiversity patterns and providing effective conservation guidelines. Field species inventories carried out by specialists (Vondrák et al. 2016, 2018; Spribille et al. 2020) as well as survey of herbaria and literature records (Isocrono et al. 2007; Himelbrant et al. 2018) are fundamental for lichen biodiversity research, sometimes triggering taxonomic advances, including the description of new species (Spribille et al. 2020; Leavitt et al. 2021; Vondrák et al. 2022). Furthermore, temporal continuity of basic biodiversity data from a given region may allow comparison of biodiversity patterns across time, to track the effect of global changes (Hauck et al. 2013).

Unfortunately, basic biodiversity data on lichens are often missing, even for relatively well-explored areas, thus hampering conservation efforts (Hunter and Webb 2002; Rubio-Salcedo et al. 2013). However, some notable exceptions exist, as in the case of the Alps, which are amongst the lichenologically best known areas of the world, thanks to their long-lasting and accurate exploration. To date, 3046 lichenised infrageneric taxa are known from the area (Nimis et al. 2018a), but this number is likely to increase with the widening of exploration and the deepening of taxonomical knowledge.

Within the Alps, the historical region of Tyrol is certainly one of the best-explored, with one of the oldest known “checklists”: in their compilative monograph “*Die Flechten (Lichenes) von Tirol, Vorarlberg und Liechtenstein*”, Dalla Torre and Sarnthein (1902) summarised a huge amount of information on the lichen biota of the Tyrolean area, mainly based on original papers, based predominantly on multiple field explorations by Ferdinand Arnold (1828–1901) and Ernst Kernstock (1852–1900). These data largely contributed to the present lichen inventory of Trentino-Alto Adige, that is the lichenologically richest region of Italy, with 1573 infrageneric taxa of lichenised fungi reported to date (Nimis and Martellos 2022).

In particular, Arnold intensely explored the area of Paneveggio and Predazzo (Arnold 1879, 1880, 1887, 1897), whose localities are famous amongst lichenologists, due to the many specimens collected there and distributed in several public herbaria, as well as to the new species described from this area. Since 1967, the area of Paneveggio was included in the Paneveggio-Pale di San Martino Natural Park, that extends south of Paneveggio to incorporate almost all the Pale di San Martino dolomitic chain and a metamorphic mountain area at the orographic right side of the Vanoi River. Since its institution, this Park has attracted lichen research thanks to the fame resulting from Arnold’s explorations. In particular, since the mid-nineties the administration of the Park promoted a new phase of exploration that focused both on lichen floristics (e.g. Nascimbene and Caniglia 2003; Thor and Nascimbene 2007) and ecology (e.g. Nascimbene and Caniglia 1997, 1999; Nascimbene et al. 2008), expanding the research effort to almost all of the protected area.

In this work, we summarise about 150 years of lichenological exploration of the Paneveggio-Pale di San Martino Natural Park, providing an updated checklist of its lichenised fungi.

## ﻿Materials and methods

### ﻿Study area

**Figure 1.**
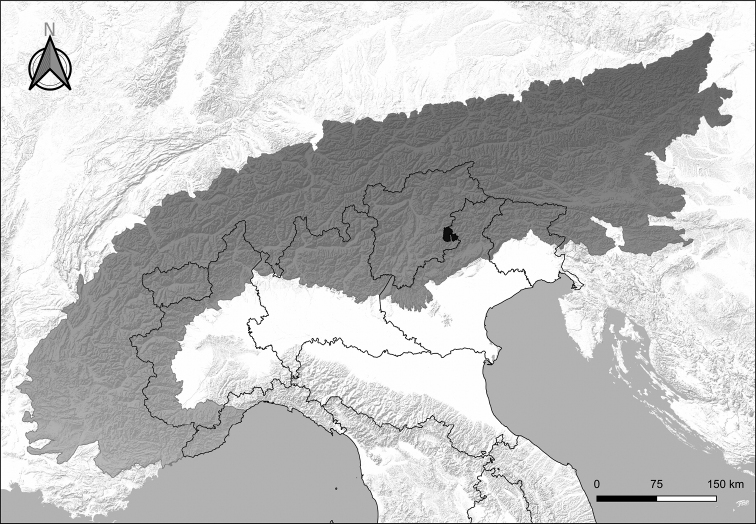
Location of the Paneveggio-Pale di San Martino Natural Park within the Alps.

The Paneveggio Pale di San Martino Natural Park, spanning an elevational gradient of about 2000 m (from 1200 m in Val Canali to 3192 m on Mt. Vezzana) and covering a surface of about 20,000 hectares, includes the typical mountain environments of the Alps, being located in the south-eastern part of the Alpine chain (Fig. 1). As a Natural Park, it includes both core areas under strict protection and buffer areas where some human activities are allowed, for example, logging, tourism and winter recreation activities.

The territory is characterised, from a geological point of view, by a high diversity of substrates. The sedimentary rocks of the Mesozoic emerge on the orographic left of the Cismon Stream, while igneous and metamorphic rocks of the Paleozoic emerge in the western part of the Park. The metamorphic unit is made up of quartz-containing phyllite and mica-schists emerging in the Scanaiol, Arzon and Tognola-Valcicolera Group. Porphyric rocks characterise the Lagorai chain, from Tognazza-Cavallazza group towards the west up to the edge of the Park, including the Bocche-Iuribrutto group. Sedimentary rocks include both well-stratified evaporitic-arenaceous formations originating between late Paleozoic and early Mesozoic (e.g. *Bellerophon* and Werfen formations) and compact dolomitic rocks (Sciliar Dolomite) which can be over a thousand metres thick. These heterogeneous sedimentary rocks characterise the landscape of the Pale di San Martino chain that reach and even exceed 3000 metres (e.g. Cimon della Pala, Mt. Mulaz, Vezzana).

The morphology of the territory influences climatic conditions: the natural barrier formed by the Pale di San Martino and Lagorai mountain ranges interrupts the flow of humid currents coming from the Adriatic Sea, determining very humid, sub-oceanic conditions on the southern slopes and cooler and drier (i.e. more continental) conditions in the northern area beyond Rolle Pass that, thus, represents a climatic border. This is reflected in differences of annual precipitation, that is higher in the southern part (i.e. San Martino di Castrozza 1550 mm/y, Val Canali 1500 mm/y) than in the northern part (i.e. Paneveggio 1180 mm/y and Predazzo 1100 mm/y). Mean annual temperature varies between 8 °C at 1100 m (e.g. Val Canali and Predazzo), 5.5 °C at 1500 m (e.g. San Martino di Castrozza) and -1 °C at 2900 m (Pale di San Martino).

The regional climate influences the distribution of vegetation types, with mixed *Abiesalba*-*Fagussylvatica*-forests in the montane belt (1000–1800 m) of the southern part and *Piceaabies*-*Larixdecidua*-*Pinuscembra* formations in the montane (1300–1800 m) and subalpine (1800–2300 m) belts of the northern part, including the famous Paneveggio Forest. In the alpine belt (2300–2700 m), primary grasslands prevail, dominated by *Carexcurvula* in the acidic part of the Park and by *Sesleriacaerulea* and *Carexsempervirens* in the carbonatic part. The nival belt (> 2700 m) hosts pioneer, discontinuous vegetation types, as in the case of chasmophytic assemblages whose composition depends on the geological substrate. Freshwater habitats (springs, rivulets, creeks) and bogs are more frequent in the porphyric-metamorphic part of the Park, while in the carbonatic part, superficial waters are rare due to Karst phenomena, being mainly related to snow-ice melting in high elevation ranges and small springs. Overall, the vascular flora is rich (about 1500 species), including several endemic taxa, such as *Campanulamorettiana*, *Primulatyrolensis*, *Saxifragafacchinii* and *Rhizobotryaalpina*, that are restricted to the Dolomites.

### ﻿The data

Between 1878 and 1886, the Bavarian lichenologist Ferdinand Arnold (1828–1901) carefully explored the region of Val di Fiemme, including the area of Paneveggio and Predazzo, for a total of 146 days of fieldwork (Arnold 1887). In summer 1884, he was supported by Hugó Lojka (1844–1887), who explored the Travignolo Valley, leading to several interesting findings (Arnold 1887). The data collected by Arnold, the oldest source on the lichen biota of the study area, are scattered in several main papers (Arnold 1869, 1875, 1879, 1880, 1886, 1887, 1889, 1893, 1896, 1897), that were later summarised in the monograph by Dalla Torre and Sarnthein (1902) (with a few genera treated by Magnus 1905). Arnold distributed a considerable number of *exsiccata* of specimens collected in the Paneveggio-Predazzo area in his “Lichenes exsiccati”, whose duplicates can be currently found in various herbaria, for example, CANB, COLO, DUKE, F, FR, GB, GZU, LD, M, NY, O, PC, S, UPS and WIS. Additional numbers were traced in, for example, Flora exs. Austro-hungarica, Lojka, Lichenotheca Universalis, Rehm - Cladoniae exs. and Zwackh - Lich. exs. Material of some more recent collections has been distributed in Plantae Graecenses. Several of Arnold’s specimens are also cited in more recent literature (Suppl. material 1). In this work, we included also Arnold’s records referring to localities that are in the surroundings of the protected area (e.g. Predazzo) for two reasons: 1) to valorise the precious work of Arnold in this region; 2) to include species potentially occurring in the protected area since they were collected on similar substrates and under comparable environmental conditions.

In the 20^th^ century, the area of Paneveggio was far less explored by lichenologists. Maria Cengia Sambo (1888–1939) published some records from the area of Passo Rolle (Cengia Sambo 1931), but specimens cited in her work are unfortunately missing to date. Later, the area was explored by Austrian lichenologists from Graz, mainly Josef Poelt (1924–1995) and Josef Hafellner, whose published and unpublished specimens are housed in GZU.

Lichenological research increased again from the late 1990s to the present and is still ongoing. Most of the records collected in this period refer to herbarium specimens and field observations by Juri Nascimbene, only a few of them having already been published (Nascimbene and Caniglia 1997, 1999, 2003; Caniglia et al. 2002; Thor and Nascimbene 2007; Nascimbene et al. 2008, 2021). The latter research, discontinuous over time, derives from an alternation of floristic and ecological studies aimed at investigating the effects of forest management on lichen diversity. While floristic studies covered the entire territory of the Park (although gaps are still present) and a wide array of substrates, the ecological studies focused on the Paneveggio Forest and included epiphytic and lignicolous lichens only. As in the case of Arnold, our checklist also includes some collections from the surroundings of the protected area.

In this work, lichenological exploration is subdivided in three main periods: 1) 19^th^ century: mainly Arnold’s collections; 2) 20^th^ century: sporadic collections mainly by lichenologists from Graz; 3) 21^th^ century (including the last five years of the previous century): mainly Nascimbene’s work.

Data were retrieved from 72 literature sources (the full list is in Suppl. material 1), eight herbaria (i.e. GB, GZU, LD, M, S, UPS, lichen herbarium of the Paneveggio-Pale di San Martino Natural Park, private lichen herbarium of Juri Nascimbene) and several field observations, mainly by Juri Nascimbene. They were organised into a georeferenced database that to date includes 7351 records. For each record, the following information was retrieved, when possible: current name (updated according to Nimis and Martellos 2022), name of the taxon in the original source, source type, locality, altitude, altitudinal belt, substrate, habitat, collection year and century, collector, identifier. Most of the historical records were incomplete, for example, by lacking detailed information on habitat and substrate. Recent collections and field observations were georeferenced and, whenever the indications of the localities allowed it (namely when a toponym, a habitat or a substrate were mentioned), historical records were georeferenced as well, with an approximation of several hundred metres, due to uncertainty. Recently collected specimens were identified by means of standard lichenological procedures, i.e. observation of morphological and anatomical features and, when needed, study of secondary metabolites by means of thin-layer chromatography in solvents A, B’ and C. For some aquatic specimens, belonging to genera *Hydropunctaria*, *Thelidium* and *Verrucaria*, molecular studies were carried out to achieve a correct identification.

Only lichenised fungi were considered; lichenicolous fungi and non-lichenised fungi usually treated by lichenologists (see Nimis 2016) are not included in this paper. Nomenclature, taxonomy and information on species’ traits refer to Nimis and Martellos (2022).

## ﻿Results

The checklist of the lichenised fungi of the Paneveggio-Pale di San Martino Natural Park includes 916 specific and infraspecific taxa (Suppl. material 1), corresponding to 58.4% of the lichen biota of Trentino-Alto Adige (Nimis and Martellos 2022), 35.2% of Italy (Nimis and Martellos 2022) and 30.1% of the Alps (Nimis et al. 2018a). Most records (4551, 731 taxa) were retrieved from literature, whereas a lesser amount refers to herbarium specimens (1325, 522 taxa) and field observations (1475, 180 taxa).

The species belong to 270 genera (most represented, with more than 20 species each: *Cladonia*, *Lecanora*, *Lecideas. lat.*, *Rhizocarpon*, *Verrucaria*, *Rinodina*; 128 genera with only one species each), 75 families (most represented, with more than 50 species each: Parmeliaceae, Lecanoraceae, Lecideaceae, Teloschistaceae, Verrucariaceae; 22 families with only one species each) and 26 orders (most represented, with more than 50 species each: Lecanorales, Verrucariales, Caliciales, Lecideales, Teloschistales, Peltigerales).

Chlorolichens are the most frequent group (93.0%), followed by cyanolichens (6.0%) and cephalolichens (1.0%); amongst chlorolichens, most have a chlorococcoid photobiont (88.3%) and only a few a trentepohlioid photobiont (4.7%). Most numerous are crustose forms (68.8%), followed by foliose (15.5%), fruticose (11.2%); squamulose (3.4%) and leprose (1.1%) forms are far less represented. Most taxa reproduce sexually (76.5%), while 23.5% reproduce asexually, mainly by soredia (17.0%), followed by isidia (4.1%) and thallus fragmentation (2.4%).

The number of subcontinental taxa is 22 (2.4%), that of suboceanic taxa 80 (8.7%), while only two taxa can be considered as oceanic (0.2%).

Four taxa are new to Italy, i.e. Fuscideamollisvar.caesioalbescens, *Hydropunctariascabra*, Protoparmeliabadiavar.cinereobadia and *Variosporapaulii*. Eighteen other taxa are new to Trentino Alto Adige, i.e. *Acarosporasphaerospora*, *Bacidinaarnoldiana*, *Chrysothrixchlorina*, *Circinariahoffmanniana*, *Dermatocarponarnoldianum*, *Gyalectaerythrozona*, Lecanorabicinctavar.bicincta, *Lecanoracaesiosora*, *Lempholemmaintricatum*, *Miriquidicaplumbea*, Myriolecisagardhianasubsp.sapaudica, *Myriolecisinvadens*, *Myriosporamyochroa*, *Parmotremaarnoldii*, Rhizocarpongeographicumsubsp.arcticum, *Sarcogyneurceolata*, *Staurothelesapaudica* and *Variosporamacrocarpa*. One species, belonging to genus *Lecanora*, still awaits a formal description as new to science (Nascimbene, pers. comm.). In previous, recent publications, several other species from the study area were recorded as new to Italy or Trentino Alto Adige (e.g. Thor and Nascimbene 2007; Nascimbene et al. 2021).

Ninety-one species are Red-listed: 62 epiphytic lichens (Nascimbene et al. 2013) and 25 terricolous lichens (Gheza et al. 2022), including four species of Cladoniasubgen.Cladina (Ravera et al. 2016).

**Figure 2.**
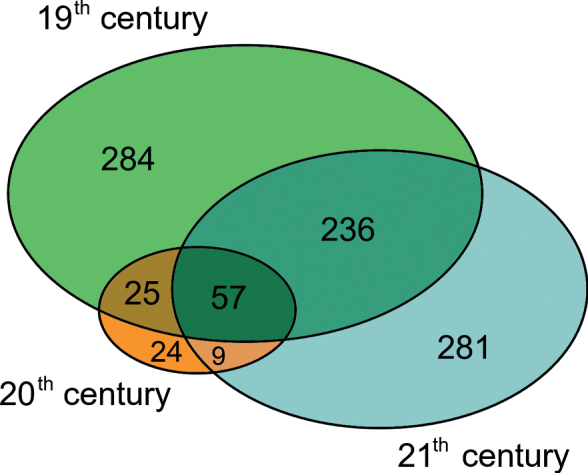
Number of lichen taxa recorded in the three exploration periods and their overlapping.

Only 57 taxa were recorded in all of the three exploration periods, whereas 271 were recorded in two of them, the largest overlap being between the 19^th^ and 21^st^ centuries, sharing 236 species (Fig. 2). Five hundred and ninety species were recorded only in one century (19^th^: 284, 20^th^: 24, 21^st^: 281). Overall, 601 taxa (3794 records) were recorded in the 19^th^ century, 116 (186 records) in the 20^th^ and 585 (3371) in the 21^st^ century.

**Figure 3.**
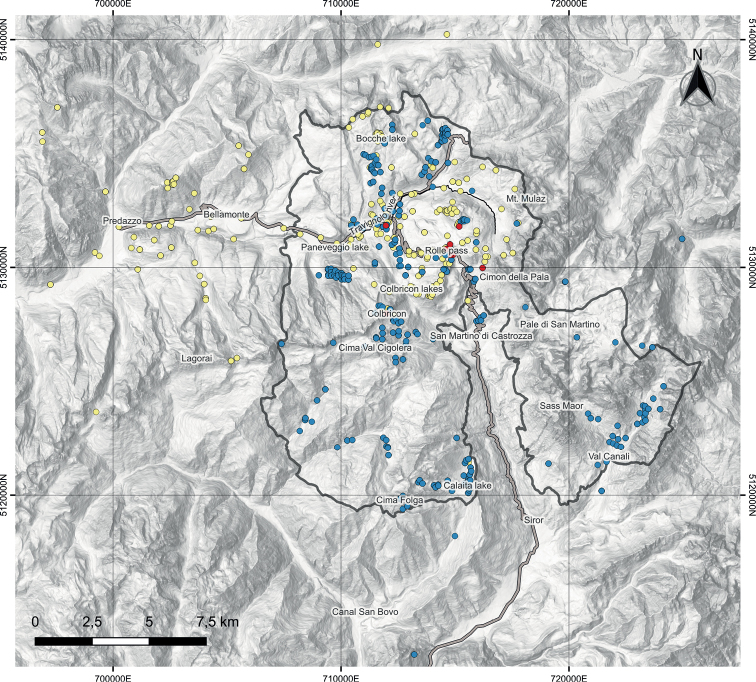
Georeferenced collection sites referred to the 19^th^ (yellow dots), 20^th^ (red dots) and 21^st^ (blue dots) centuries; the continuous black line indicates the borders of the Paneveggio-Pale di San Martino Natural Park.

The spatial distribution of historical (Arnold’s) and recent (20^th^ and 21^th^ centuries) records reflects the exploration history, with Arnold’s localities concentrated in the northern part of the protected area (and its surroundings; Fig. 3) and recent records also scattered in the southern part of the Natural Park, both in the dolomitic and metamorphic areas where, however, some gaps still remain.

**Figure 4.**
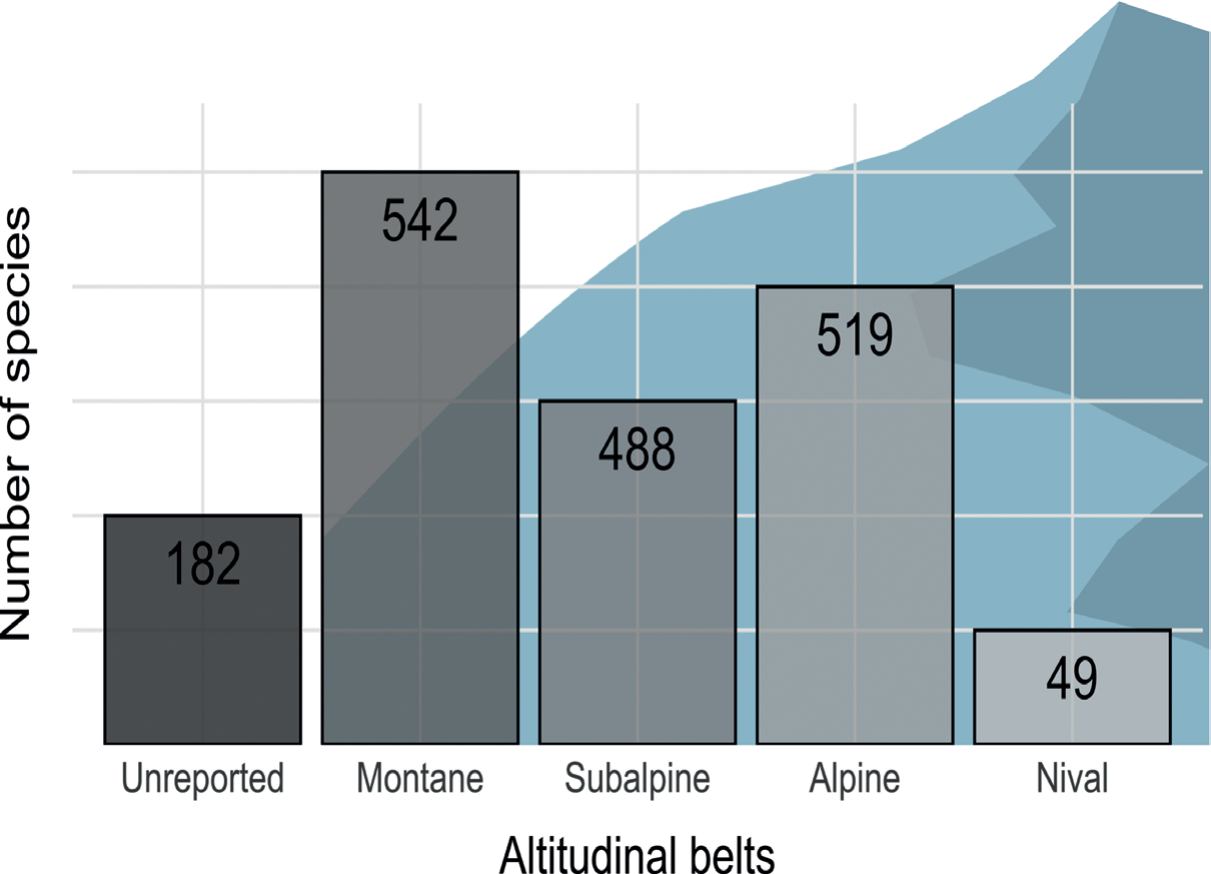
Number of lichen taxa in each altitudinal belt.

The montane belt was the most explored, with 2654 records of 535 taxa, followed by the subalpine, (2296 records of 476 taxa) and the alpine belts (1852 records of 514 taxa) (Fig. 4). The nival belt was the less explored, with 109 records of 49 taxa (Fig. 4).

**Figure 5.**
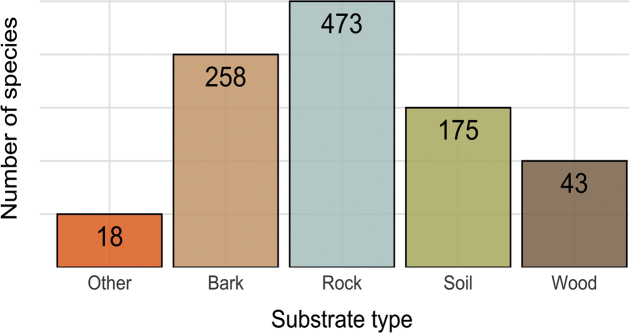
Number of lichen taxa on the main substrate types.

The highest number of records is from rocks (2351 records, 458 taxa), followed by bark (2003 records, 257 taxa) and soil (665 records, 116 taxa) (Fig. 5). Other substrates, such as deadwood, are less represented. Information about rock type, tree species and soil type was not always available. Amongst saxicolous lichens, most records are from magmatic and metamorphic siliceous rocks (1368 records, 287 taxa), while carbonatic rocks are poorer (873 records, 214 taxa). Epiphytic lichens were mainly collected on *Piceaabies* (133 taxa, 730 records), followed by *Abiesalba* (61, 174), *Larixdecidua* (48, 527), *Pinuscembra* (34, 161), *Alnusincana* (24, 52), *Rhododendronferrugineum* (24, 48) and *Fraxinusexcelsior* (19, 19). Terricolous lichens were mainly from acidic soil (53 taxa), with 25 taxa from carbonatic soil.

## ﻿Discussion

The Paneveggio-Pale di San Martino Natural Park can be considered as a hotspot of both lichenological research, with more than 150 years of exploration, and of lichen diversity. Almost one third of the lichen biota of both the Alps and Italy occurs in this area, whose surface is ca. 0.06% of their total surface area. This highlights its importance for lichen conservation and lichenological research, with several regionally and nationally new taxa, the occurence of species that still await formal description or of taxa that are known from this area only, as in the case of *Thelidiumpaneveggiensis*. Moreover, lichen diversity is at least 60% of that of vascular plants, indicating that lichens strongly contribute to the biodiversity of the protected area.

This level of knowledge of the lichen biota is rare in protected areas of the European Alps. Arnold himself stated that, thanks to the repeated and careful investigations he carried out “from the valleys to the highest heights”, the upper Val di Fiemme could be considered as the lichenologically best known area of Tyrol at the time (Arnold 1887). A similar situation is perhaps that of the High Tauern National Park, in which over 1100 species have been recorded since the times of Arnold (Türk 2016) on an area which is, however, larger by a factor of ten. In the Italian Alps, other checklists are available, as in the case of a sector of the Stelvio National Park (Nascimbene et al. 2012) or for the Sciliar Natural Park in South Tyrol (Nascimbene 2008), but these are far less exhaustive and the number of species will certainly increase with further exploration. In the case of lichens, not easily detectable and often with a rarefied distribution (Nimis et al. 2018b), it is difficult to provide exhaustive checklists. However, when exploration is concentrated on relatively small and environmentally heterogeneous areas, the number of species can be surprisingly high (Vondrák et al. 2022). At a national level, in the absence of comparable knowledge on other protected areas, the Paneveggio-Pale di San Martino Natural Park is certainly a priority area for lichen conservation, which should be amongst its main management aims.

This small Natural Park has a great climatic, geological and orographical heterogeneity that likely enhances lichen diversity (Vondrák et al. 2022). For example, Passo Rolle, located in the central part of the study area, is a boundary between oceanic (south) and continental (north) climates, as well as a geological and tectonic boundary. The climatical heterogeneity determines the occurrence of many species with different phytoclimatic affinities, i.e. 22 subcontinental and 82 suboceanic/oceanic taxa. Geological diversity as well plays an important role in shaping and enriching lichen diversity, at least with regard to saxicolous and terricolous species: the checklist includes many specialists of either siliceous or carbonatic rocks and soils, whose co-occurrence in the study area is allowed by the high variety of rock types. Finally, the wide altitudinal range offers favourable conditions for montane, subalpine, alpine and nival species. This also implies different tree species available for epiphytic lichens along the gradient, from broadleaved forests at lower altitudes to coniferous stands in the highest forested belts.

The other component of this lichen hotspot is its exploration history, starting from the 19^th^ century. It should be noticed that, at the times of Arnold, explorations were much more difficult: although he spent a long time in the study area, the investigations carried out in the last decades covered an overall longer timespan and also took into account several areas not explored by Arnold. Nevertheless, a high number of taxa was recorded only either by Arnold or by Nascimbene, but it is hard to say whether the species recorded only in the 19^th^ century could actually have disappeared today. In some cases, the lack of recent records is probably due to merely overlooking the widespread and common taxa in recent surveys, as in the cases of *Athalliapyracea*, *Circinariacalcarea* and *Physconiagrisea* that surely still occur. It is also difficult to understand how several widespread or locally common species that likely already occurred at the times of Arnold went unnoticed in historical times and were recorded only in the 21^st^ century, as in the cases of *Athalliacerinella*, *Cladoniasymphycarpa*, *Everniaprunastri* and *Lecidellaelaeochroma*. On the other hand, some species were recorded only in recent times, because they were described recently (e.g. *Absconditellalignicola*, *Anaptychiabryorum*, *Caliciumpinicola* and *Variosporapaulii*) or were recognised later as independent taxa (e.g. *Cetreliacetrarioides*, *C.monachorum* and *C.olivetorum*). Even when the same locality was visited across the three periods, as in the case of Mt. Castellazzo, the overlapping of records was relatively low, differences being mainly related to poorly detectable species, such as small crustose and endolithic lichens and perhaps also the the bias related to the effect of different collectors. Under these circumstances, the checklist is likely more an image of lichen diversity taken with a long exposure time rather than a generalised framework for directly assessing changes of the lichen biota across time, that can be only achieved with resampling of small and clearly localised plots. Only in the case of some easily-detectable species, sensitive to environmental changes (e.g. *Nephromalaevigatum*, *Stictafuliginosa* and *Usnealongissima*) that were not recorded in recent years, we could hypothesise that they actually disappeared due to global changes (i.e., climate, land-use, forest management).

## ﻿Conclusions

The checklist of the lichens of the Paneveggio-Pale di San Martino Natural Park contributes to a better knowledge of the lichen biota at a broader level than a mere local checklist. It has: (1) a biogeographical value, including a high number of records useful to better elucidate the distribution of many rare and/or poorly known taxa; and (2) a value for biodiversity conservation, providing a framework on which further research can be based. Such detailed floristic information is useful to plan new explorations for assessing the occurrence of the rarest species, which is of paramount importance for planning future conservation actions. Focusing on this topic with a targeted sampling could help to understand the effects of environmental changes in the last 150 years (Hauck et al. 2013), including increased human impact and the ongoing climate change.

Last but not least, this checklist is a remarkable demonstration that even the best-studied areas can still reveal many novelties and should not be considered as “accomplished missions”, but should be monitored continuously.

## Supplementary materials

Supplementary material 1 A hotspot of lichen diversity and lichenological research in the Alps: the Paneveggio-Pale di San Martino Natural Park (Italy) Data type occurrences https://binary.pensoft.net/file/778315This dataset is made available under the Open Database License (http://opendatacommons.org/licenses/odbl/1.0/). The Open Database License (ODbL) is a license agreement intended to allow users to freely share, modify, and use this Dataset while maintaining this same freedom for others, provided that the original source and author(s) are credited.Juri Nascimbene, Gabriele Gheza, Peter O. Bilovitz, Luana Francesconi, Josef Hafellner, Helmut Mayrhofer, Maurizio Salvadori, Chiara Vallese, Pier Luigi Nimis
